# Aerial Images and Convolutional Neural Network for Cotton Bloom Detection

**DOI:** 10.3389/fpls.2017.02235

**Published:** 2018-02-16

**Authors:** Rui Xu, Changying Li, Andrew H. Paterson, Yu Jiang, Shangpeng Sun, Jon S. Robertson

**Affiliations:** ^1^Bio-Sensing and Instrumentation Lab, College of Engineering, University of Georgia, Athens, GA, United States; ^2^Plant Genome Mapping Laboratory, Department of Genetics, University of Georgia, Athens, GA, United States

**Keywords:** cotton, flower, bloom, unmanned aerial vehicle, point cloud, convolutional neural network, phenotyping

## Abstract

Monitoring flower development can provide useful information for production management, estimating yield and selecting specific genotypes of crops. The main goal of this study was to develop a methodology to detect and count cotton flowers, or blooms, using color images acquired by an unmanned aerial system. The aerial images were collected from two test fields in 4 days. A convolutional neural network (CNN) was designed and trained to detect cotton blooms in raw images, and their 3D locations were calculated using the dense point cloud constructed from the aerial images with the structure from motion method. The quality of the dense point cloud was analyzed and plots with poor quality were excluded from data analysis. A constrained clustering algorithm was developed to register the same bloom detected from different images based on the 3D location of the bloom. The accuracy and incompleteness of the dense point cloud were analyzed because they affected the accuracy of the 3D location of the blooms and thus the accuracy of the bloom registration result. The constrained clustering algorithm was validated using simulated data, showing good efficiency and accuracy. The bloom count from the proposed method was comparable with the number counted manually with an error of −4 to 3 blooms for the field with a single plant per plot. However, more plots were underestimated in the field with multiple plants per plot due to hidden blooms that were not captured by the aerial images. The proposed methodology provides a high-throughput method to continuously monitor the flowering progress of cotton.

## Introduction

Improving cotton yield is one of the main objectives in cotton breeding projects and cotton growth and production management. Yield can be defined as the product of the number of bolls produced per unit area and the mass of lint per boll (a common measure of boll size). Cotton yield is associated with many physiological variables and environmental factors. However, an increase in cotton yield is generally associated with an increase in the number of bolls regardless of genotype or environment (Wells and Meredith, 1984; Pettigrew, 1994). Flower and boll retention will affect the final number of bolls produced. Therefore, processes that affect flower and boll retention will have a significant impact on yield.

Flowering is important to cotton yield because pollinated flowers form cotton bolls. Within a given genotype, seasonal flower production per unit area is more closely related to yield than boll retention percentage (Heitholt, 1993, 1995). Since a cotton flower is white (cream-color for some upland germplasms and yellow for Pima) on the first day it opens and turns pink within 24 h, it is unlikely to mistake an old bloom for a new bloom on separate days. Therefore, if bloom counts are obtained daily then the seasonal total counts can be calculated. The flowering time (the time of the first flower opening) and the peak flowering time can be determined accordingly, both of which are critical to production management. The timing and duration of the flowering stage also reflect the difference in growth habits of different genotypes, which can help breeders select specific genotypes, for example, short-season or long-season genotypes. Although manual counting is perhaps the simplest and most reliable way to count flowers, it is tedious and inefficient and requires a massive amount of labor, which is not practical for large fields.

Imaging methods can improve the efficiency of manual counting. Many studies have been done on flower detection and classification using color images (Adamsen et al., 2000; Siraj et al., 2010; Hsu et al., 2011; Biradar and Shrikhande, 2015; Seeland et al., 2016; Thorp et al., 2016). Flowers usually have distinct color and texture from the background; therefore, traditional image processing methods such as color and texture analysis can be used to segment flower pixels (Thorp et al., 2016). The number of flowers can be calculated using morphological operations on the segmented flower pixels or correlated with flower pixel percentage (Biradar and Shrikhande, 2015; Thorp et al., 2016). Flower features can be extracted from flower images and are used to recognize flower species (Hsu et al., 2011). Machine learning techniques can be used to classify different flower types, which could be useful to determine the age of cotton flowers based on differences in color and shape (Seeland et al., 2016). Deep learning methods such as the convolutional neural network (CNN) have been demonstrated to be effective in recognizing flower species (Liu et al., 2016). CNN showed advantages over traditional machine learning methods because it does not require extraction of image features.

Although imaging methods are efficient in detecting flowers, the image collection throughput limits its usage in agriculture because agriculture usually deals with large fields. To improve the data collection throughput, the use of an unmanned aerial vehicle (UAV) is usually preferred over a ground vehicle or a robot because a UAV can provide superior data collection speed and larger spatial coverage. UAVs also do not interact with the plants, so constant data collection will not cause soil compaction and plant damage, which can happen when using a ground vehicle. Although many researchers have used UAVs for agriculture studies in recent years, only a few used UAVs to count flowers for crops. For example, aerial multispectral imaging has been used to calculate flower fraction in oilseed rape and monitor the peach flower (Fang et al., 2016; Horton et al., 2017). However, those studies only segmented flowers from the canopy rather than counting the flowers. To our knowledge, no study has been done to count flowers using aerial images. The main reason for this is the low image resolution compared to images taken from ground platforms, making image processing challenging.

In this paper, we aimed to develop a methodology for counting cotton blooms using aerial color images. The overall objective of this paper was to develop a data processing pipeline to detect and count the number of newly opened cotton flowers using aerial images. Specific objectives were to: (1) build and train a CNN to classify flowers, (2) construct dense point clouds from raw images and evaluate the quality, (3) develop an algorithm to register flowers, and analyze its accuracy and efficiency using simulated data, and (4) evaluate the performance of the data processing pipeline compared with manual counting. To avoid ambiguity, we used the term bloom to refer to a newly opened flower to distinguish it from its other growth stages.

## Materials and methods

### Test fields

**Figure 1:**
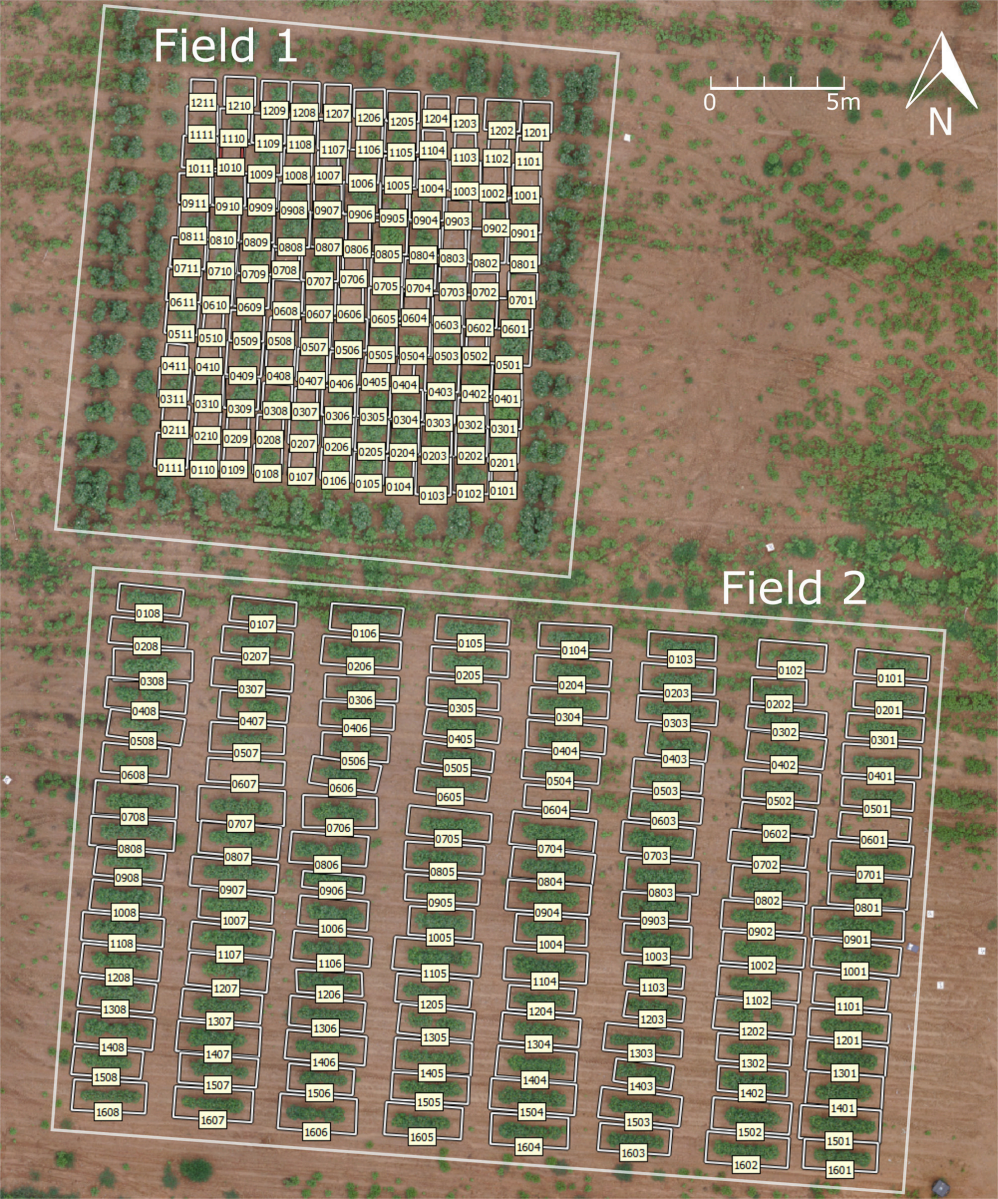
Plot layout of the two test fields.

Two test fields were used, both of which were located in the Plant Science Farm in Watkinsville, GA (33°43′37.80″N, 83°17′57.52″E) (Figure 1). Field 1 had 132 plots with a single cotton plant in each plot, arranged in 12 rows and 11 columns with a 1.5 m (5 ft) distance in both row and column directions. The cotton in field 1 was planted on May 25, 2016. Field 2 had 128 plots of cotton, arranged in 16 rows and 8 columns (Figure 1). Each plot was 3 m (10 ft) long and 1.5 m (5 ft) wide, with a 1.8 m (6 ft) alley between each plot. Fifteen cotton plants were planted in each plot with equal spacing of 0.15 m (6 inches) on June 13, 2016. The plant density varied because of the different number of plants germinated, resulting in some empty plots in field 1 and field 2. The number of germinated plants in each plot for field 2 was recorded 2 weeks after planting (June 8, 2016) to be used to calculate the average number of blooms for each plant.

### Data collection

Aerial color images of the test fields were collected using an octocopter (s1000+, DJI, China) with a color camera (Lumix DMC-G6, Panasonic, Japan) on August 12, August 19, August 26, and September 9, 2016 (Table 1). The color camera was directly mounted on the bottom of the drone with the lens facing downward. An inertial measurement unit (IMU) with GPS was mounted on the drone to record the location and orientation of the camera. Raspberry Pi 2 was used to trigger the camera and record the IMU/GPS measurement. During the flight, the camera took images at a frequency of 1 Hz. The exposure time and aperture were manually set for the color camera according to the light conditions of the field, and auto-settings were used for other parameters. The drone was flown at a height of 15 m above ground level (AGL) to reduce the ground sample distance (GSD). One flight was used to collect data from both of the test fields.

**Table 1:** Data collection summary.

**Date**	**Time (p.m.)**	**Flight height (m)**	**Flight speed (m/s)**	**Focal length (mm)**	**Ground pixel size (mm)**
8/12/2016	12:09	15	2.5	18	3.17
8/19/2016	1:43	15	1.5	20	2.23
8/26/2016	1:35	15	3	20	2.69
9/9/2016	1:12	15	2.5	22	2.36

The number of blooms in each plot was manually counted as a reference for the image method. To count the blooms in each plot, only white flowers were counted in each plot on the same day that aerial images were collected. Although manual counting is reliable overall, it is possible for some blooms to be counted multiple times or not counted. The judgment of whether a flower is white is subjective and varies from person to person, which could cause human counting errors. For field 1, blooms in all of the plots were counted on all 4 days. For field 2, blooms were counted in all plots on August 12, and in 32 random plots on the other 3 days.

### Data processing pipeline

**Figure 2:**
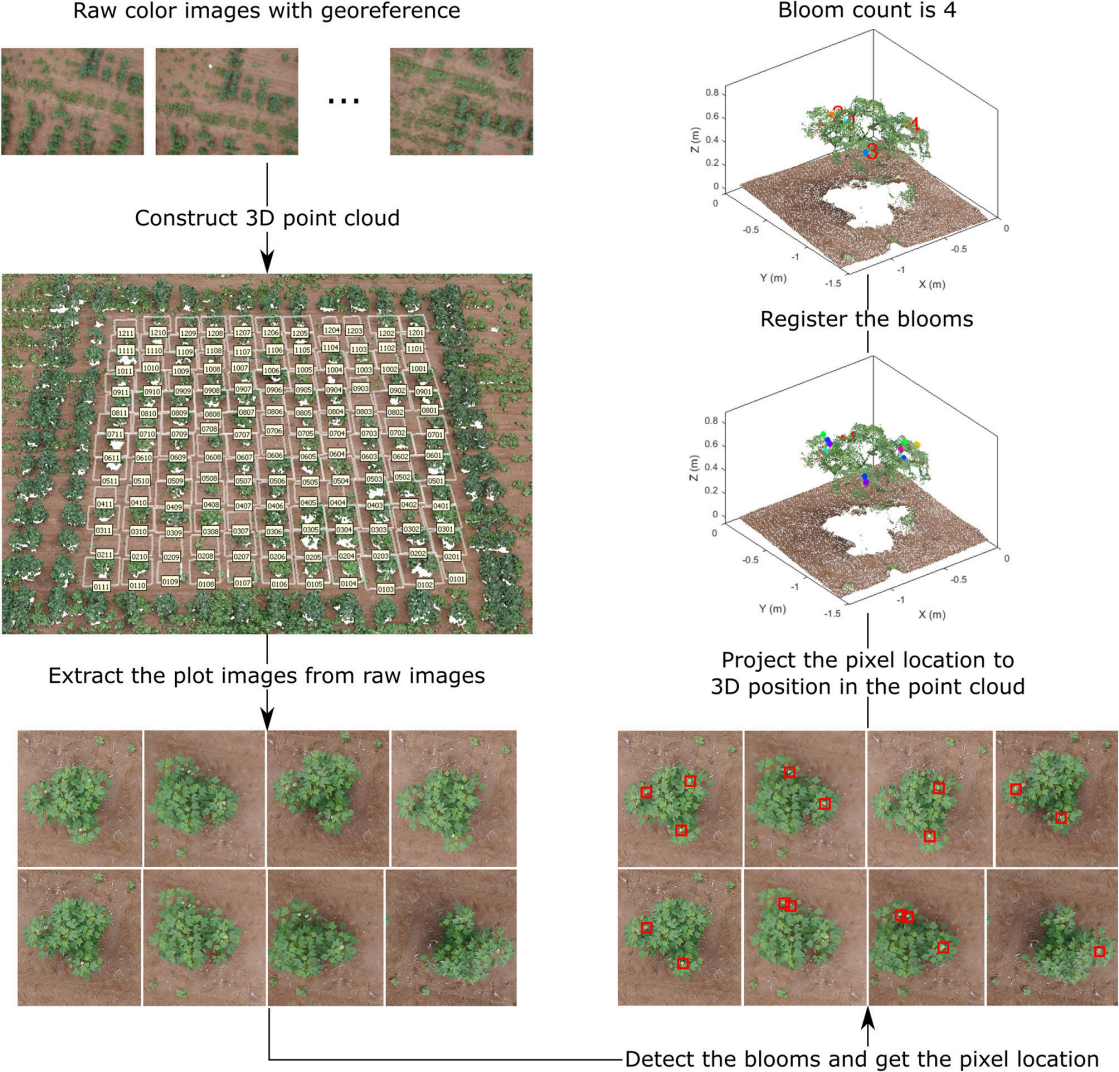
Overall flowchart of the bloom detection algorithm, demonstrating the output of each step for field 1.

The overall data processing pipeline for bloom counting can be divided into four key steps: (1) dense point cloud generation, (2) plot images extraction, (3) bloom detection, and (4) bloom registration (Figure 2). The final output of the pipeline is the bloom count for each plot. Other information such as bloom position can be obtained too.

#### Dense point cloud generation

A dense point cloud of each test field was generated in PhotoScan (PhotoScan Professional 1.3.1, Agisoft, Russia) using the raw color images and the IMU/GPS measurements. The high accuracy and reference preselection settings were used for photo alignment, the high-quality setting for dense point cloud build, and default for other settings. After photo alignment, PhotoScan constructed a tie point cloud—often called a sparse point cloud in other software—from the feature points detected in the images. Before constructing the dense point cloud, the tie point cloud was used to calculate the accuracy of the dense point cloud.

After building the dense point cloud, it was found that field 1 was not fully covered by the images collected on August 19 and those uncovered plots were excluded from further data analysis. Additionally, some plots had an incomplete point cloud for the canopy due to low side-overlap on August 19, August 26, and September 9, when the focal length was enlarged to reduce the ground pixel size. Point cloud coverage, which is the percentage of the plot constructed with valid point cloud, was used to evaluate the completeness of the point cloud. The plots with point cloud coverage less than 80% were excluded from further analysis. The method to calculate point cloud coverage is explained in section Quality of the dense point cloud.

#### Plot images extraction

The main purpose of the plot images extraction step is to export plot images from the raw images such that one plot image contains only one plot or part of the plot. Since one plot can be imaged several times, one plot has multiple plot images from different raw images. This is helpful for detecting blooms inside the canopy because different raw images provide views of each plot from different angles, which greatly improves the chance of one bloom being imaged compared to the ortho-image, which only shows the top view of the canopy. To extract the plot images, plot boundaries were first manually drawn for each plot and stored as quadrangle shapes in PhotoScan. Then the four vertices of each shape were projected to each raw image to get the pixel location of the vertices in each image. One image was considered to cover part of the plot if two or three vertices were in the image (pixel location is within the boundary of the image), and the whole plot if four vertices were in the image. The pixel location of the four vertices was recorded and plot images were extracted from the raw image accordingly. The step was processed in PhotoScan using built-in Python functions. The image file name, plot number, and pixel locations of the four vertices were saved in a text file and imported into MATLAB (MATLAB 2017a, MathWorks, USA) to extract plot images from raw images.

#### Bloom detection

The bloom detection process contains two steps. The first step is to screen out the locations that most likely contain cotton blooms based on the fact that cotton blooms usually have the highest pixel intensity because of their white color. The plot image was first transferred into CIELAB color space and then the screening was performed using the L channel. A threshold of 0.75 on the normalized L channel (normalized by the maximum value in L channel) was used to extract bright objects. The number of pixels for each object was counted and noisy small objects less than 15 pixels were removed. Objects separated by a distance of less than the diameter of a flower—which is about 20 pixels for our dataset—were combined into one object because some flowers were split into two objects by the leaves. Images with a size of 36 by 36 pixels around the center of the remaining objects were extracted from the plot image. Those images were treated as potential bloom images.

The second step is to classify the potential bloom images into bloom and non-bloom class using the pre-trained CNN (detailed information on the CNN and training process is described in section Convolutional neural network). After classification, the images classified as bloom class were kept and the pixel location of those images in the raw color images was recorded.

#### Bloom registration

Since the same bloom can be detected in several images, it is necessary to register the bloom before counting the blooms to prevent counting the same bloom more than once. Registration methods based on image features were not useful because very few features could be detected from the bloom images due to the resolution limit. Therefore, we first projected the pixel location of each detected bloom into the 3D location in the dense point cloud, and then clustered the blooms based on the locations because the same bloom should have the same 3D location in the dense point cloud. The projection was done in PhotoScan. Because each pixel in the image has a corresponding image ray, any point in the image ray will appear as the same pixel in the image, and the 3D location of that pixel in the point cloud is the intersection between the image ray and the point cloud. If there is no intersection between the image ray and the point cloud, PhotoScan will return the closest point to the image ray.

Due to the accuracy of the point cloud and pixel location, the 3D location of the bloom may deviate from the true location, which increases the chance of incorrect bloom registration solely based on the location. However, since multiple blooms detected in the same image are different blooms and cannot be in the same cluster, the bloom registration based on the 3D position can be generalized as a constrained clustering problem as follows:

*Given the set of data points and the set of their corresponding classes, form the data points into clusters so that no data points in the same class will be in the same cluster and the distance between any two clusters are not smaller than a threshold*.

In the case of bloom registration, the data point is the bloom position and the class is the image number to indicate in which image the bloom was detected. Although existing constrained clustering algorithms can solve the problem, most of the algorithms are for general clustering problems and are not efficient for this specific problem. Therefore, inspired by hierarchical clustering, a deterministic clustering algorithm was designed specifically for the bloom registration problem. The algorithm initializes with each data point as one cluster. The algorithm involves cluster selection and merging. First, for each cluster *i*, a set of clusters, S, was selected from all of the clusters such that no data points in S had the same class as any data point assigned to cluster *i*. Second, the distance between cluster *i* and every cluster in S was calculated and cluster *i* was merged to the closest cluster in S if their distance was smaller than the threshold λ. The algorithm repeats the selection and merging until no merging happens. The algorithm has a similar effect as the regular hierarchical clustering algorithms when the distance between two clusters is measured using Equation (1).

(1) $$dist\left(\mu_{i,}\mu_{j}\right)=\{\begin{array}{cc} dist^{\prime }\left(\mu_{i,}\mu_{j}\right), & if\;E_{i}\cap E_{j}=\emptyset \\ +\infty , & otherwise \end{array}$$

where μ_*i*_ and μ_*j*_ are the center of the cluster *i* and *j*, $dist^{\prime }\left(\mu_{i,}\mu_{j}\right)$ is the distance metric used in the hierarchical clustering algorithms, and *E*_*i*_ and *E*_*j*_ are the set of classes of the data points assigned to cluster *i* and *j*. Compared to the regular hierarchical clustering algorithms, the efficiency of our algorithm is improved because the distance calculation is only performed on a subset of the clusters.

Euclidean distance was used to measure the cluster dissimilarity. However, because of the large error of the 3D position projection on the z-axis, the Euclidean distance was modified by adding a weight term on the z-axis to adjust the influence of z-axis on the distance. The weighted Euclidean distance between *a* = (*a*_*x*_, *a*_*y*_, *a*_*z*_) and *b* = (*b*_*x*_, *b*_*y*_, *b*_*z*_) was calculated using Equation (2). The final program used 0.5 for *w*.

(2) $$dist\left(a,b\right)=\sqrt{{\left(a_{x}-b_{x}\right)}^{2}+{\left(a_{y}-b_{y}\right)}^{2}+w^{2}{\left(a_{z}-b_{z}\right)}^{2}}$$

**Table:** **Bloom registration algorithm**.

**Input:** Set of data points $D\;=\;{\left\{x_{i}\right\}}_{i}^{n}$, class $E={\left\{e_{i}\right\}}_{i}^{n}$, and distance threshold λ.
**Result:** The assignment variables for the data points, *z* = [*z*_1_, *z*_2_, …, *z*_*n*_].
**Initialization:** each point is assigned as one cluster, *z*_1_ = 1, *z*_2_ = 2, ⋯ , *z*_*i*_ = *i*, ⋯ , *z*_*n*_ = *n*
**while** *not converged* **do**
**for** *i* = 1 : *n* **do**
Find cluster *i* and its center **μ**_*i*_
**if** cluster *i* exists **then**
S←∅;
Given the current assignment of points, find the set of classes of the data points assigned to cluster *i*,
*E*_*i*_ = {*e*_*k*_ ∈ *E*|*z*_*k*_ = *i*};
**for** *j* = 1 : *n* **do**
Find cluster *j* and its center **μ**_*j*_
**if** cluster *j* exists **then**
Given the current assignment of points, find the classes of the data points assigned to cluster *j*,
*E*_*j*_ = {*e*_*k*_ ∈ *E*|*z*_*k*_ = *j*};
**if** *E*_*i*_ ∩ *E*_*k*_ = ∅ **then**
// Add the current cluster to the selected clusters.
$S\leftarrow \{S\cup \mu_{k}\};$;
**end**
**end**
**end**
**end**
$[d_{min},i_{min}]\leftarrow min\{dist(\mu_{i},s_{1}),\cdots ,dist(\mu_{i},s_{\left|\;S\right|})\};$;
**if** *d*_*min*_ < λ **then**
//Merge cluster *i* with *i*_*min*_ and update the assignment variables;
Assign *i*_*min*_ to the assignment variables for the data points in cluster *i*;
**end**
**end**
**for** *k* = 1 : *n* **do**
Given the current assignment of points, find the set of points assigned to cluster *k*, $k,D_{k}=\;\{x_{i}\;\in D|z_{i}=k\}:$
**if** $|D_{k}|>\;0$ **then**
$\mu_{k}\leftarrow \frac{\sum_{x_{i}\in \;D_{k}}x_{i}}{\left|D_{k}\right|}$
**else**
Remove the cluster and apply the changes to the related variables;
**end**
**end**
**end**

### Convolutional neural network

**Figure 3:**
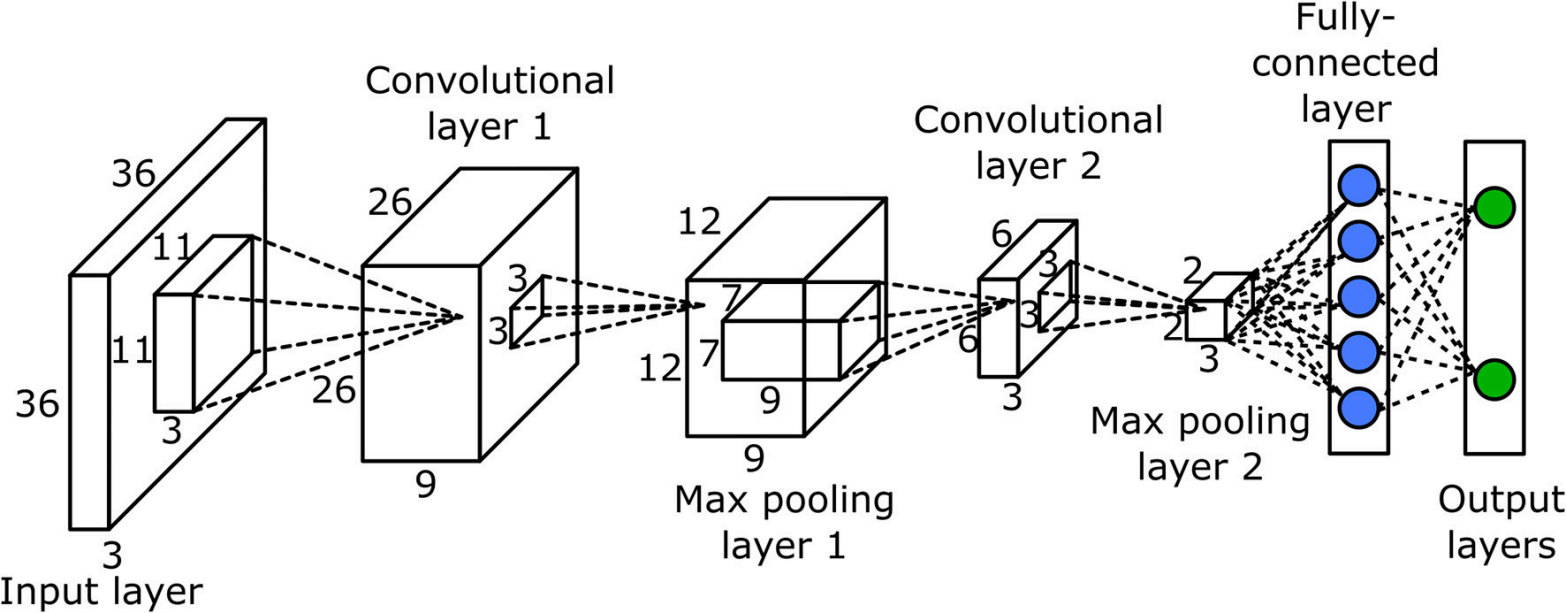
Structure of the convolutional neural network.

The CNN was used to classify potential bloom images. The network had seven layers, one input layer, two convolutional layers, two max pooling layers, and two fully connected layers (Figure 3). Since the classification was performed on the potential bloom images, the potential bloom images were first extracted from individual data sets and manually labeled into bloom and non-bloom classes. Then the labeled potential bloom images were used to construct the image database. In total, ~14,000 images for the bloom class and ~60,000 images for the non-bloom class were extracted. Two thirds of the bloom images (roughly 9,000 images) and the same number of non-bloom images were randomly selected as training samples. The remaining images were used as testing samples. The CNN was trained for 30 epochs. The learning rate was set to 0.01 at the first epoch and decreased by a factor of 10 every 10 epochs. The mini batch size was set to 256. The regularization factor was set to 0.01 to prevent overfitting.

### Quality of the dense point cloud

The quality of the dense point cloud of the canopy greatly affects the accuracy of the 3D position of each bloom, and thus the clustering results, and ultimately the bloom numbers. The quality of the dense point cloud can be evaluated from two aspects—the accuracy and the completeness of the dense point cloud.

The position accuracy of each point in the point cloud is difficult to calculate because the geometry of the canopy is unknown. Instead, the projection and reprojection error were calculated for the tie points to estimate the overall accuracy of the dense point cloud. The projection error was calculated as the distance between the projection of the feature points to the point cloud and their corresponding tie points. The reprojection error was calculated as the pixel distance between the projection of the tie points on the images and their original corresponding feature points. The projection error was analyzed on each single axis (easting, northing, and elevation) and the summation of three axes.

The completeness of the dense point cloud quantifies how completely the point cloud represents the real object or scene, and can be measured by the density of the point cloud. The completeness of the point cloud on the easting-northing plane was calculated since the camera only captures the top view. First, the point cloud was rasterized into a 2D elevation map using grid steps of 1.3 cm without interpolating the empty cells. The elevation map was divided into each plot. For each plot, the ground surface was calculated using the Maximum Likelihood Estimation SAmple Consensus (MLESAC) (Torr and Zisserman, 2000). The plot was divided into ground and canopy using the elevation map based on the distance to the ground surface with a threshold of 0.1 m. The point cloud coverage (PCC) for the canopy is defined in Equation (3).

(3) $$PCC=\frac{Area\;of\;the\;canopy}{Area\;of\;the\;canopy+Area\;of\;the\;empty\;cells}$$

The area of the empty cells was included in the denominator because the empty cells were usually part of the canopy. The PCC estimates the completeness with which the canopy is represented by the dense point cloud, which can greatly affect the bloom registration result.

### Evaluation of the bloom registration algorithm

To evaluate the efficiency and accuracy of the bloom registration algorithm, artificial data was generated in three steps. First, a data point with three dimensions was generated by randomly drawing integers from 0 to 9 for each axis. This step was repeated until 125 different data points were generated. The same class number was assigned to the 125 data points to simulate that they were from the same image. Second, the first step was repeated 10 times, generating in total 1,250 data points. A new class number was assigned to the 125 data points at each repeat to simulate that the data points from each repeat were from different images. The data points with the same coordinates were set in the same cluster, which is the true clustering result. Third, a random error that followed $N\left(0,\frac{\sqrt{3}}{3}\sigma \right)$ was added to each axis of each data point, thus the position error (vector summation of the errors of the three axis) followed N(0,σ). Different σ was used to simulate the noise level of the 3D position of the bloom. The 1,250 data points were clustered using the bloom registration algorithm and compared with the true clustering result.

Since the clustering result will depend on the noise level of the data and threshold for the clustering algorithms, 10 different σ values from 0.1 to 0.5 with an interval of 0.05, and 5 threshold values were tested. The threshold values were σ, 1.5σ, 2σ, 2.5σ, and 3σ and they covered from 68 to 99.7% of the position error. To acquire statistics of the result, the third step of generating artificial data was repeated 10 times to generate 10 sets of artificial data for each noise level and threshold. The mean and standard deviation of the clustering error and runtime on the 10 data sets were analyzed. The misclustering rate was defined using equation 4.

(4) $$misclustering\;rate=\frac{number\;of\;misclustered\;points}{total\;number\;of\;points}$$

Since it is difficult to relate the clustering result with the ground truth, the misclustered points were considered as the points that were in the same cluster from the clustering result but not in the same cluster from the ground truth. The number of misclustered points was calculated with two approaches. The first approach—referred to as misclustering rate by reference—used the true clustering result as the ground truth, and the second approach—referred to as misclustering rate by result—used the clustering results as the ground truth. The first approach was good at evaluating the clustering error when the clustering algorithm over clustered the data points, while the second approach was suitable for under-clustering errors.

## Result

### Quality of the dense point cloud

**Figure 4:**
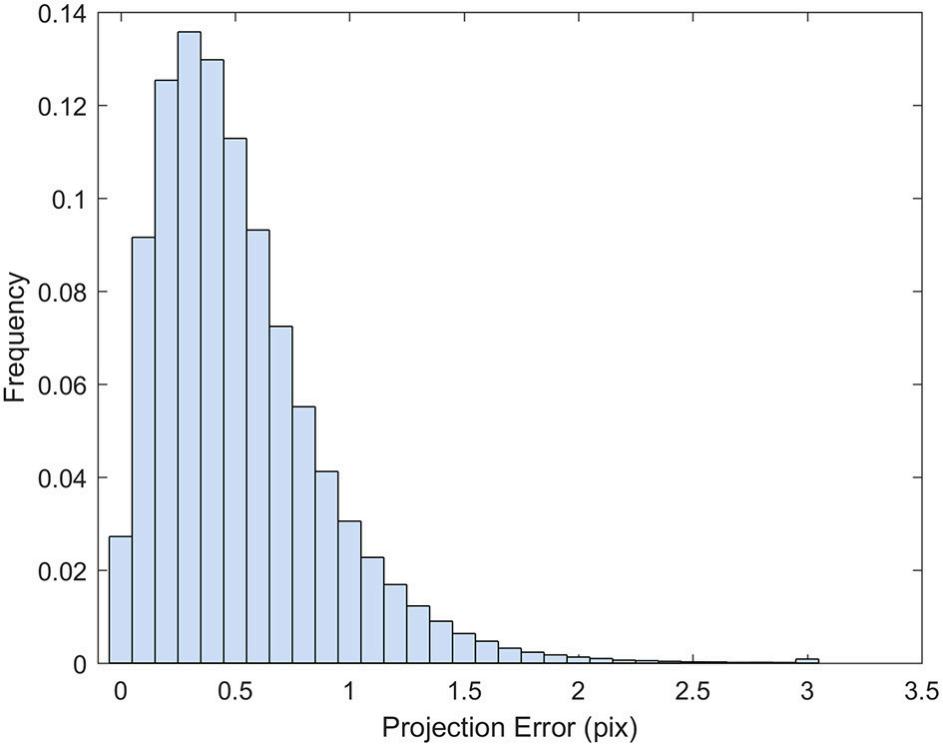
Histogram of the reprojection error for the tie points generated from 8/12/2016 dataset.

**Figure 5:**
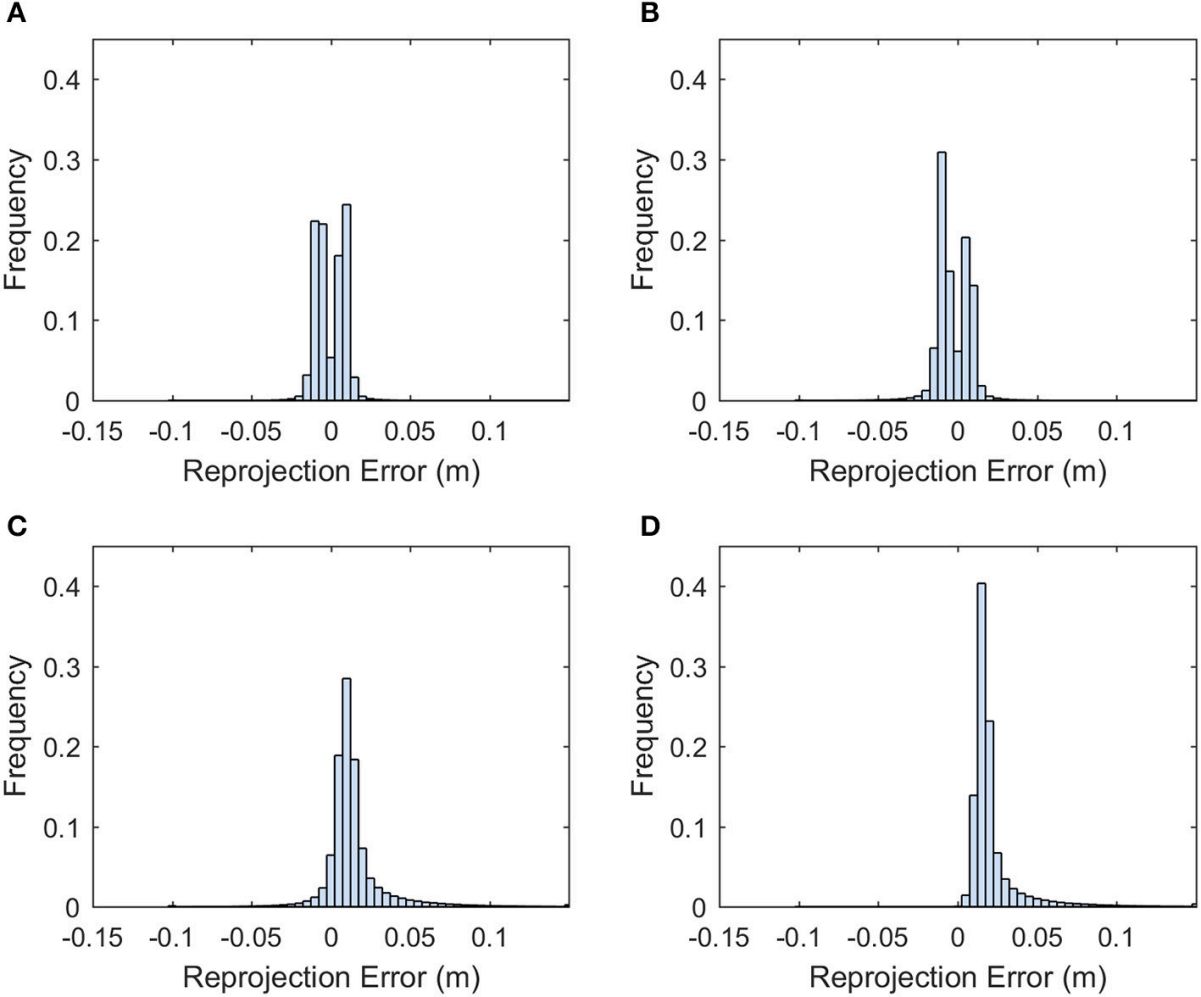
Histogram of the projection error for the tie points generated from the 8/12/2016 dataset. **(A)** Error histogram for easting. **(B)** Error histogram for northing. **(C)** Error histogram for elevation. **(D)** Error histogram for the summation of the three axes.

The point cloud accuracy was analyzed using the dataset collected on 8/12. More than 99% of the reprojection errors of the tie points were less than 1.7 pixels and only a few were larger than 2 pixels (Figure 4). The mean reprojection error was 0.5 pixels. The tie points had overall larger projection error on the elevation than the easting and northing (Figure 5). The mean projection error for the easting and northing was 0 m because the positive and negative errors were canceled out, whereas the mean projection error for the elevation was 0.014 m, which is reasonable since the depth generated from multi-view stereo is usually the least accurate. The elevation also had larger variation than the easting and northing. The mean projection error for the three axes combined was 0.022 m, and 99% of the errors were between 0 and 0.127 m (Figure 5D). The large projection error on the elevation validates the rationale to assign a weight to the elevation when calculating the cluster distance in bloom registration.

In the event of an incomplete dense point cloud for the canopy, some blooms may not be able to get 3D point positions and thus cannot be counted. If the blooms equally distribute over the canopy, the point cloud coverage can be considered as the probability of a bloom having a valid point in the dense point cloud. Assuming a canopy has *n* blooms and point cloud coverage of *p*, then *k* (*k* = 1, 2, …, *n*) blooms not having valid points (which cannot be counted using the imaging method) follow a binomial distribution *B*(*n*, 1 − *p*). The mean count error is *n*(1 − *p*) and the relative error is (1 − *p*). Therefore, the point cloud coverage should be close to 1 to make the counting error small.

**Figure 6:**
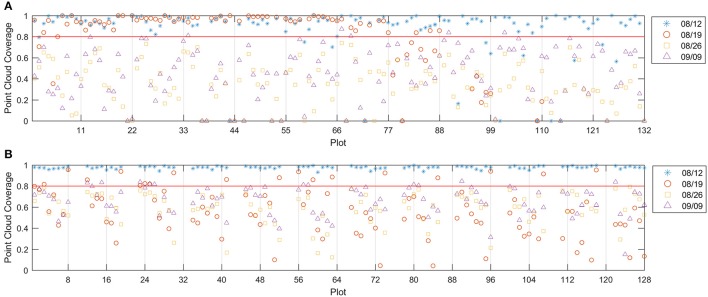
Point cloud coverage for field 1 **(A)** and field 2 **(B)**. The red line indicates the 0.8 threshold.

Figure 6 showed that the 8/12 dataset had good point cloud coverage (> 0.9) on most of the plots for both test fields. However, the other three datasets showed low point cloud coverage on both fields, except that the 8/19 dataset had some plots with good point cloud coverage. The low point cloud coverage was mainly due to the insufficient image side-overlap (<60%); the PhotoScan was unable to construct valid 3D points for areas that were covered by less than three images. Therefore, increasing the image overlap can improve the point cloud coverage. The depth filter inside the PhotoScan removed noisy points due to the movement of the plants and image noise, which was another reason for the low coverage. To achieve low image counting error, the plots with point cloud coverage lower than 0.8 were excluded from data analysis, which removed 16 plots in field 1 for the 8/12 dataset, 18 plots in field 1 and 103 plots in field 2 for the 8/19 dataset, all the plots in field 1 and 112 plots in field 2 for the 8/26 dataset, and 127 plots in field 1 and 97 plots in field 2 for the 9/9 dataset.

### Training result of the convolutional neural network (CNN)

**Figure 7:**
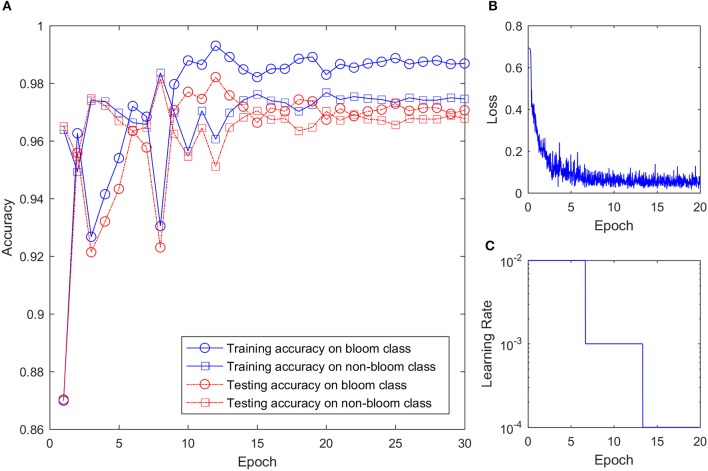
CNN training setting and result. **(A)** Training and testing accuracy of the CNN on two classes over training epoch. **(B)** Training loss over epoch. **(C)** Learning rate over epoch.

With more than 28,000 training images and mini batch size of 256, the CNN was trained about 114 iterations every epoch. Because of the large training sample size and relatively simple structure, the training process converged quickly and the training accuracy reached 0.94 in the first epoch. As shown in Figure 7, the training accuracy for bloom and non-bloom class increased in the first few epochs. The training accuracy for the non-bloom class reached a maximum on the 8th epoch, and the training accuracy for the bloom class reached a maximum on the 12th epoch. The accuracy for both classes decreased after reaching the maximum value, fluctuated a bit, but then reached a stable accuracy with small variation after 20 epochs. The training loss decreased quickly over the first few epochs, which indicates the quick convergence of the training. After 10 epochs, where the learning rate changed from 0.01 to 0.001, the training loss kept decreasing but the change rate was small. After 20 epochs, the training loss reached the minimum with a certain level of oscillation, which indicates that the training process should stop at the 20th epoch because no further improvement of the CNN can be gained from the last 10 epochs. Therefore, the training result at the 20th epoch was used to classify the potential bloom images. The testing accuracy was smaller than the training accuracy for both classes after 7 epochs but the difference between them was less than 0.01, which showed the CNN worked well on both the training and testing sets.

**Figure 8:**
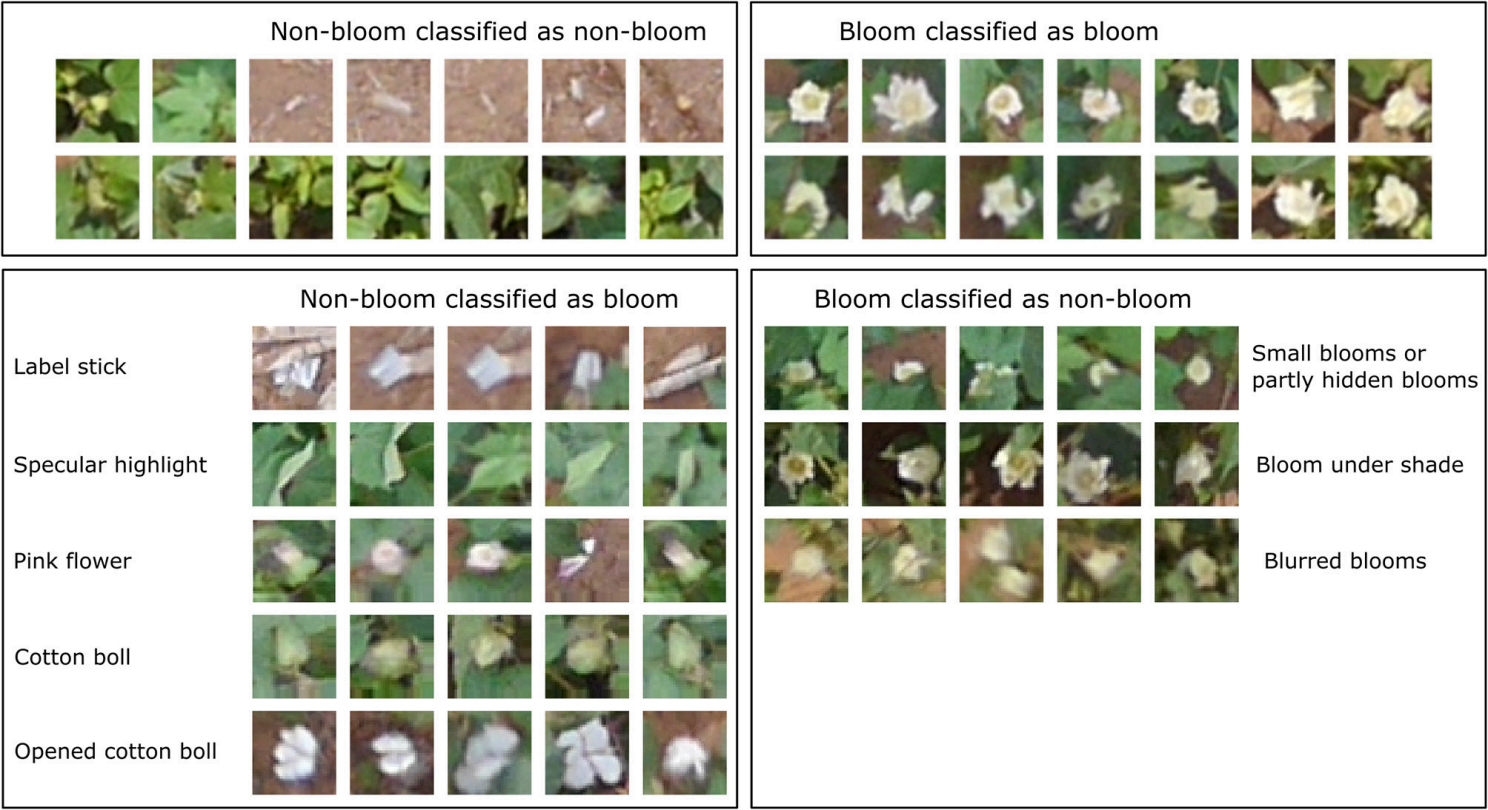
Example images of the classification result.

The classification result for each dataset using the trained CNN varied due to the different lighting conditions and flowering stages (Table 2). The classification result showed high precision (>0.9) for both classes across all datasets. However, the recall (true positive rate) for the bloom class was low for the 8/19 and 9/9 datasets. The CNN can cause overestimation or underestimation when comparing the number of predicted blooms and the number of actual blooms. For example, in the 8/12 dataset, the number of predicted bloom images was 789, but the actual number of bloom images was 719, thus the classification result overestimated the number of blooms by 10%. Similarly, the classification result overestimated the number of blooms by 23 and 29% in the 8/19 and 9/9 datasets, and underestimated the number of blooms by 2% in the 8/26 dataset. Objects such as cotton bolls, specular highlights on leaves, and pink flowers were misclassified as blooms because their shape and color appeared like a bloom in the aerial image due to the limited resolution (Figure 8). The misclassified blooms caused by objects (e.g., label sticks) on the ground could be removed based on the height from the ground, but the misclassified blooms caused by the plants (e.g., leaves, pink flowers, and cotton bolls) were difficult to eliminate using the height. Small blooms or partly hidden blooms can easily be misclassified as non-blooms because of their size. Blooms in the shade can also be misclassified as non-blooms because their intensity is reduced. Including those misclassified images into the training set may further improve the CNN performance.

**Table 2:** Classification result of the potential bloom images extracted from individual dataset for field 1.

	**8/12/2016**				**8/19/2016**				**8/26/2016**				**9/9/2016**			
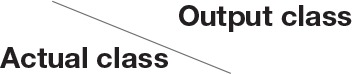	**Bloom**	**Non-bloom**	**Precision**	**Recall**	**Bloom**	**Non-bloom**	**Precision**	**Recall**	**Bloom**	**Non-bloom**	**Precision**	**Recall**	**Bloom**	**Non-bloom**	**Precision**	**Recall**
Bloom	709	10	0.90	0.90	813	37	0.96	0.78	731	82	0.90	0.91	558	28	0.95	0.74
Non-bloom	80	4876	0.98	0.997	234	23208	0.99	0.998	68	8543	0.99	0.99	191	3946	0.95	0.99

### Efficiency and accuracy of the bloom registration algorithm

**Figure 9:**
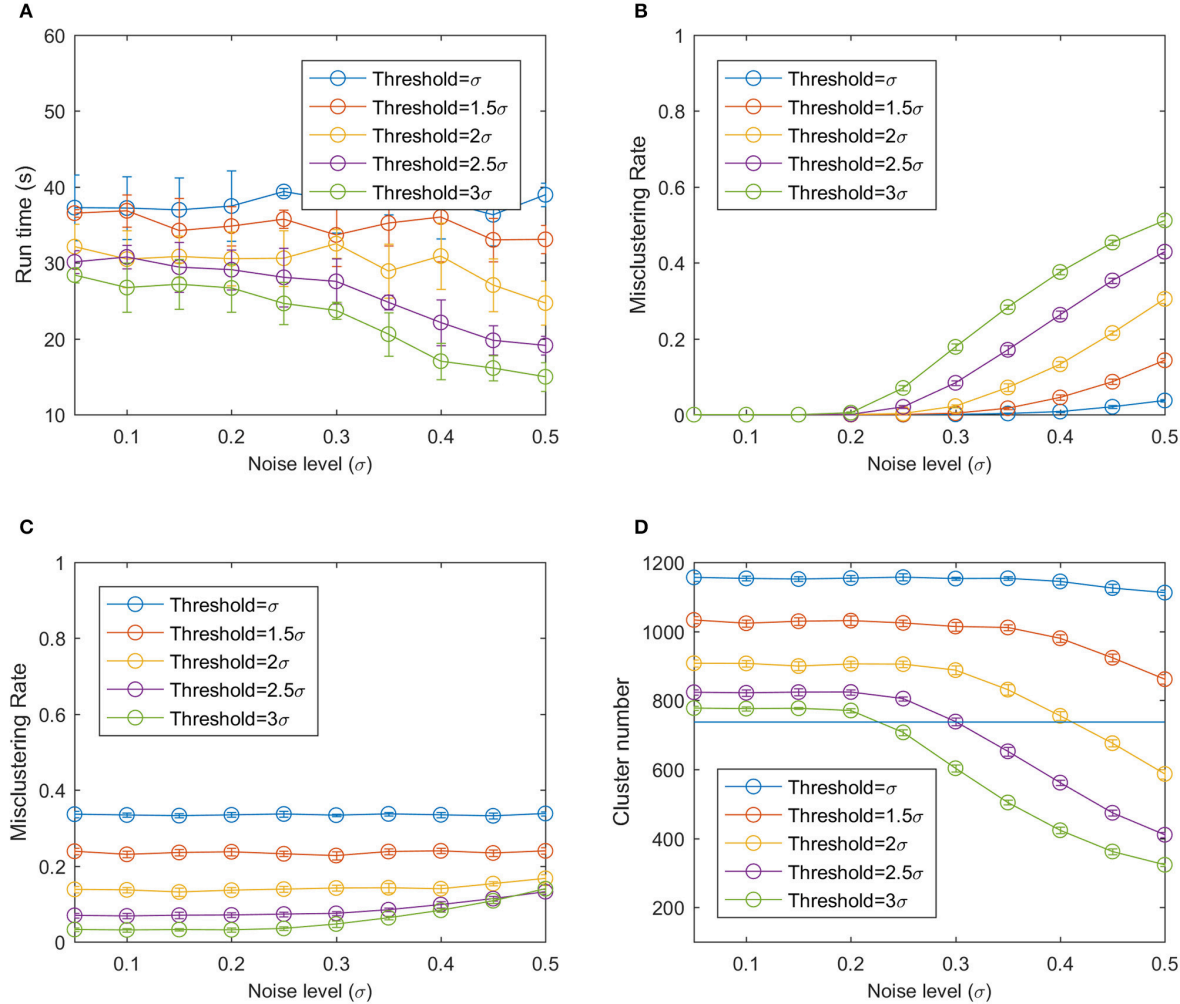
**(A)** Run time of the bloom registration algorithm on the simulation data. **(B)** Misclustering rate by result. **(C)** Misclustering rate by reference. **(D)** Number of clusters using modified hierarchy clustering. The horizontal line is the true number of clusters.

To cluster the 1,250 data points, the bloom registration algorithm used 13.3 to 48.4 s, which is 0.0106 to 0.0387 s per data point (Figure 9A). Using a larger threshold and larger noise level reduced the runtime because they resulted in a smaller number of clusters and thus the number of distance calculations. The bloom registration algorithm produced a near-zero misclustering rate by result at smaller noise level no matter what threshold was used because the distance between two reference clusters was still larger than the spread of the data points due to the noise (Figure 9B). As the noise level increased, the misclustering rate by result increased and larger threshold values had larger misclustering rates. This was mainly because of over-clustering when the noise of the points became large enough that certain portions of two clusters overlapped with each other and the algorithm could cluster them into one cluster. The misclustering rate by reference did not change significantly as the noise level increased (Figure 9). Smaller thresholds had larger misclustering rates by reference.

At low noise level, the under-clustering takes the main effect because the bloom registration algorithm can split one cluster into smaller clusters at low noise level, generating a larger number of clusters compared to the real cluster number (Figure 9D). As the noise increased, a smaller number of clusters than the real number of clusters was generated, resulting in over-clustering. The bloom registration algorithm had a smaller cluster number with larger threshold at the same noise level, indicating that it is more prone to under-clustering but less prone to over-clustering.

### Bloom count result

**Figure 10:**
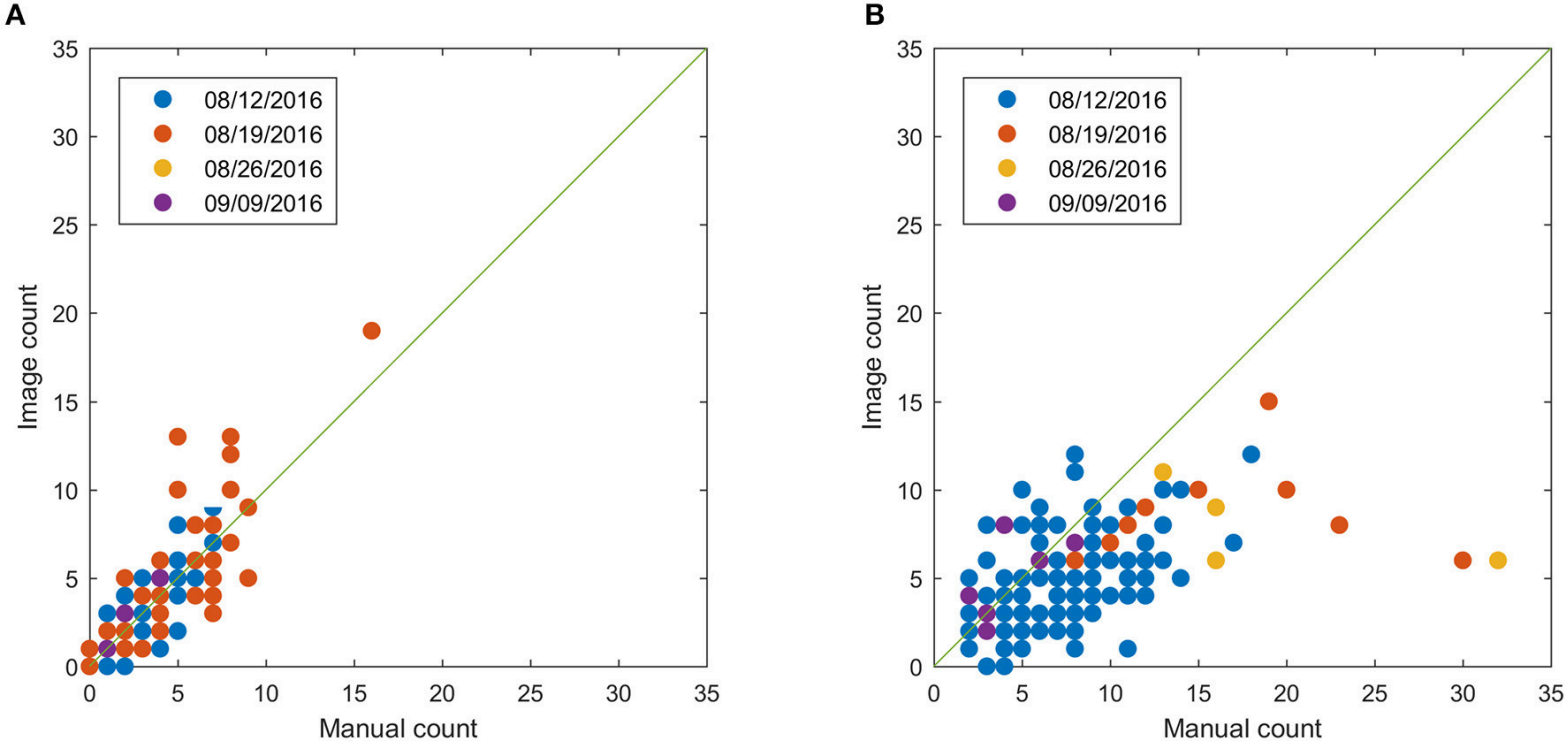
Comparison between image count and manual count for field 1 **(A)** and field 2 **(B)** after removing plots with point cloud coverage less than 0.8.

**Figure 11:**
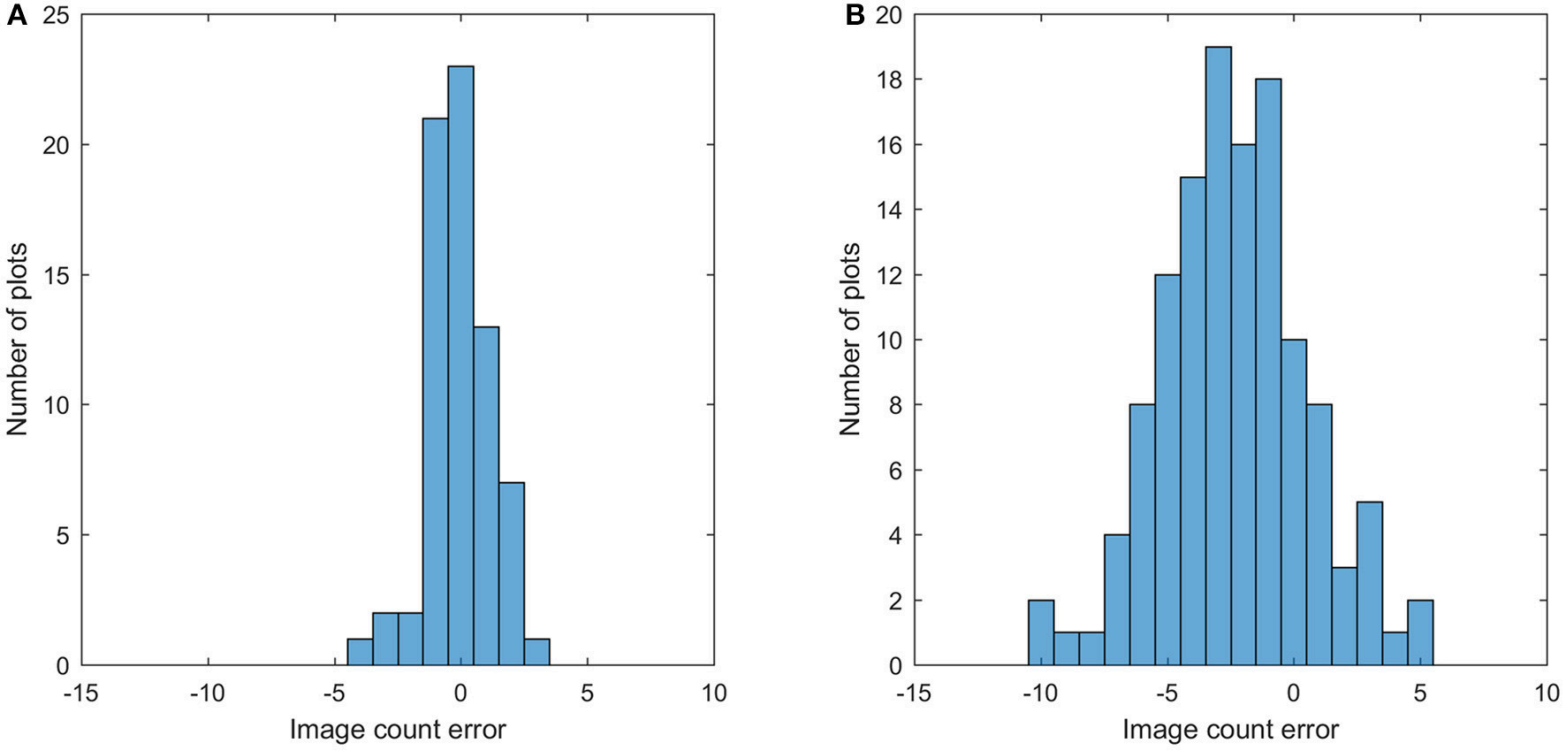
Histogram of the image count error for field 1 **(A)** and field 2 **(B)**.

This section shows the bloom count results after removing the plots with point cloud coverage less than 0.8. The image count and manual count had the same trend for both fields (Figure 10). The error of the image count was between −4 and 3 for field 1, and the between −10 and 5 for field 2, showing that field 2 had more underestimated plots than field 1 (Figure 11). This may be due to the single plant per plot layout in field 1. The single plant per plot layout allows the plant to be seen from all directions without being blocked by other plants in the plot. Therefore, blooms in field 1 were more likely to be captured in the aerial images.

**Figure 12:**
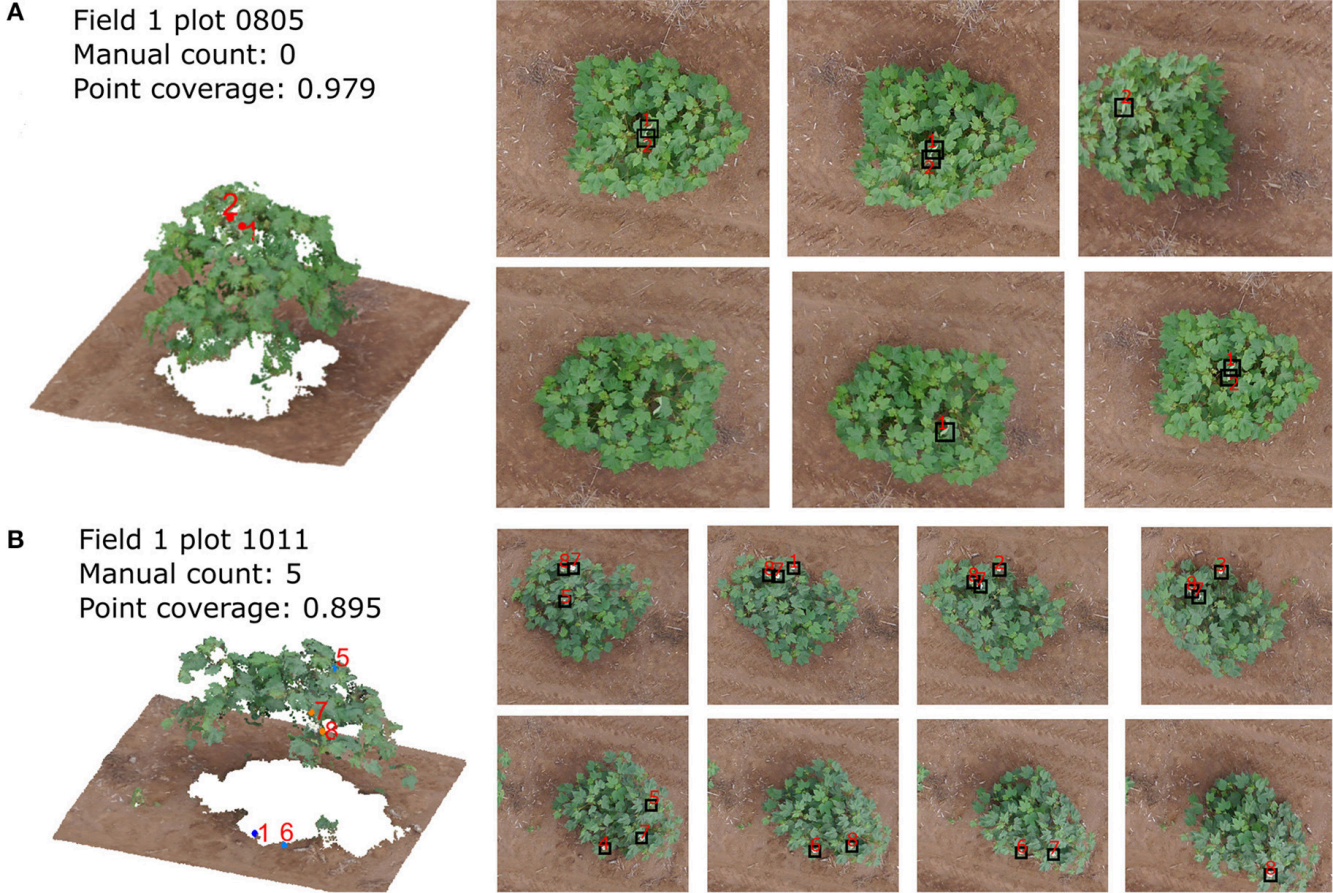
The bloom detection results for plot 0110 **(A)** and plot 1011 **(B)** in field 1 on 8/12/2016 dataset. The left images show the point cloud and detected blooms and right images show the corresponding blooms in the raw images.

The overestimation was caused for two major reasons. One reason is the classification error of the CNN. For example, some leaves with specular highlights were classified as blooms (Figure 12A). The other reason is the quality of the point cloud, which causes the error of the bloom's 3D position and thus make the bloom registration incorrect (the same bloom from different images was registered as different blooms) (Figure 12B). The underestimation was caused by hidden blooms that were not shown in the aerial images, or blooms that were shown in the images but were not classified correctly by the CNN.

**Figure 13:**
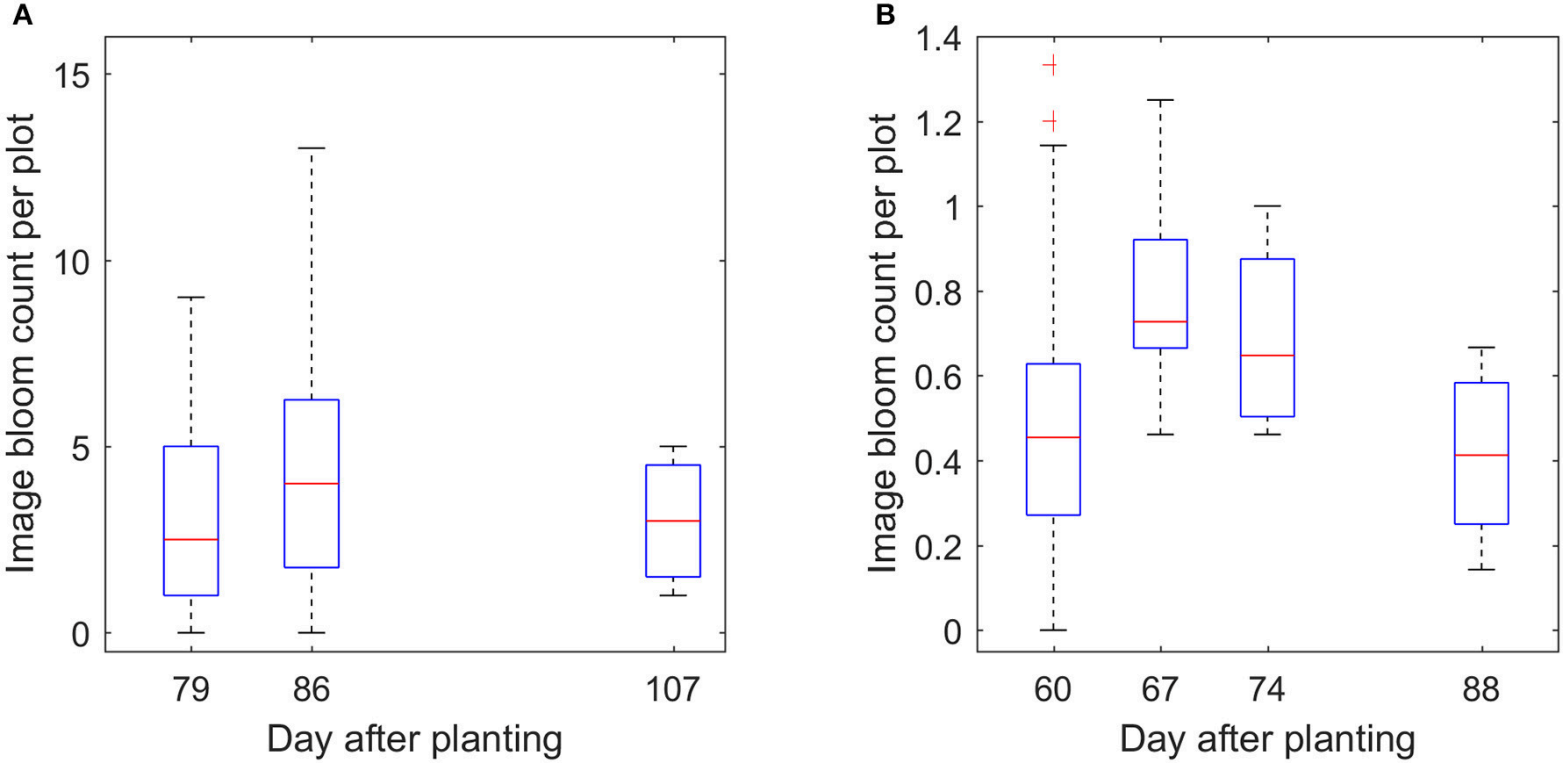
Boxplot of the image count per plot over time for field 1 **(A)** and field 2 **(B)**.

With enough datasets, it is possible to monitor the development of the flowers over time, which is one of the advantages of the proposed method. Figure 13 demonstrates the trend of the bloom development. It shows that the number of blooms was low at the early flowering stage, reached the peak in the middle flowering stage, and then decreased at the late flowering stage. This trend is also consistent with the trend from manual counting (Figures S1–S5).

## Discussion

This paper demonstrated a high-throughput methodology to count cotton blooms using aerial color images. Continuously monitoring the cotton blooms over time can provide information about the cotton growth status, such as the flowering time and peak flowering time, which can be used for production management and yield estimation. It is also helpful for breeding programs to identify short season or long season genotypes.

Unlike sorghum or corn, whose flowers open at top of the stem, cotton flowers open from the bottom and progress up the plant. At the early cotton flowering stage, blooms at the bottom can be covered by the leaves and may not be able to be captured by the aerial images. Therefore, it is expected that image counting can underestimate the real bloom count. Instead of using the ortho-images which only take the top view of the canopy, we utilized all the raw images that take different views of a plot to get the bloom count in order to improve the underestimation to a certain extent. The inability to detect hidden blooms inside the canopy from aerial images is the major limitation of the proposed methodology. This issue could be addressed by using oblique aerial images or ground side-view images. The underestimation could be improved at the middle and later flowering stages since the flowers that open at the middle and top of the canopy are more likely to be imaged in the aerial images.

Besides the inability to detect hidden flowers, the proposed bloom counting method can generate errors from two aspects: the bloom detection error and the bloom registration error. The bloom detection error was affected by the image quality, the threshold to select the potential bloom images, and the accuracy of the CNN. The image quality was affected by the illumination condition at the time of data collection. Ideally, the best image quality can be obtained when the illumination is uniform from all directions so there are no shadows or specular highlights. The ideal condition is hard to obtain in the field, but the approximate ideal condition can be obtained on a cloudy day. When the sunlight directly shines on the canopy without being scattered by clouds, shadows and specular highlights can be found in the aerial images, which cause non-uniformed intensity changes of the plot. Those changes will affect the selection of the potential bloom positions.

The threshold to select the potential bloom positions was arbitrary and mainly based on the images. A high threshold can eliminate some blooms that have low intensity due to shadows. A low threshold can include more non-bloom objects (such as the specular highlights), which can increase the processing time and be misclassified by the CNN. The classification of the potential bloom images relied on the CNN but misclassifying a bloom image as a non-bloom image affected the result differently from misclassifying a non-bloom image. When a bloom was classified as a non-bloom in one image, it was possible to be correctly classified as a bloom in other images given a different perspective of the flower; therefore, this flower could be included in the final result. However, when a non-bloom image was classified as a bloom, this false bloom was counted and there was no approach to remove it in the current method. The training samples for the CNN were selected from images collected on only 4 different days, so the trained CNN may not be suitable for data collected in different growth stages, especially when the color of the cotton leaves changes over the season. Therefore, including more training samples from different dates over the growth season could improve the accuracy and robustness of the CNN. The different appearances of the cotton flower caused by genotype differences—for instance, the Pima cotton has yellow bloom in contrast with white bloom for some upland cotton varieties—should also be considered when constructing the training samples.

The accuracy of the bloom registration was affected by the 3D position of the bloom and the registration algorithm. The 3D position of the bloom was affected by the pixel location and the dense point cloud. Therefore, the accuracy of the dense point cloud has an important impact on the accuracy of a bloom's 3D position, and thus affects the bloom registration results. A larger error of the 3D position can cause the bloom registration to under-cluster or over-cluster the blooms. Under-clustering can count the same bloom more than once and over-clustering may count several blooms as one bloom, making the bloom count unreliable. The completeness of the point cloud also affects the registration result. If the dense point cloud cannot cover the whole canopy, some blooms that are detected in the images may not have a valid projection on the dense point cloud. Those blooms will not be registered and underestimation of the bloom count will occur. Therefore, adequate image overlap is critical in data collection to capture the whole canopy. Increasing the image overlap can improve the completeness, but also requires more data collection and processing time. Oblique imagery can also improve the completeness of the point cloud by providing more views of a plot and potentially can image more occluded flowers.

**Figure 14:**
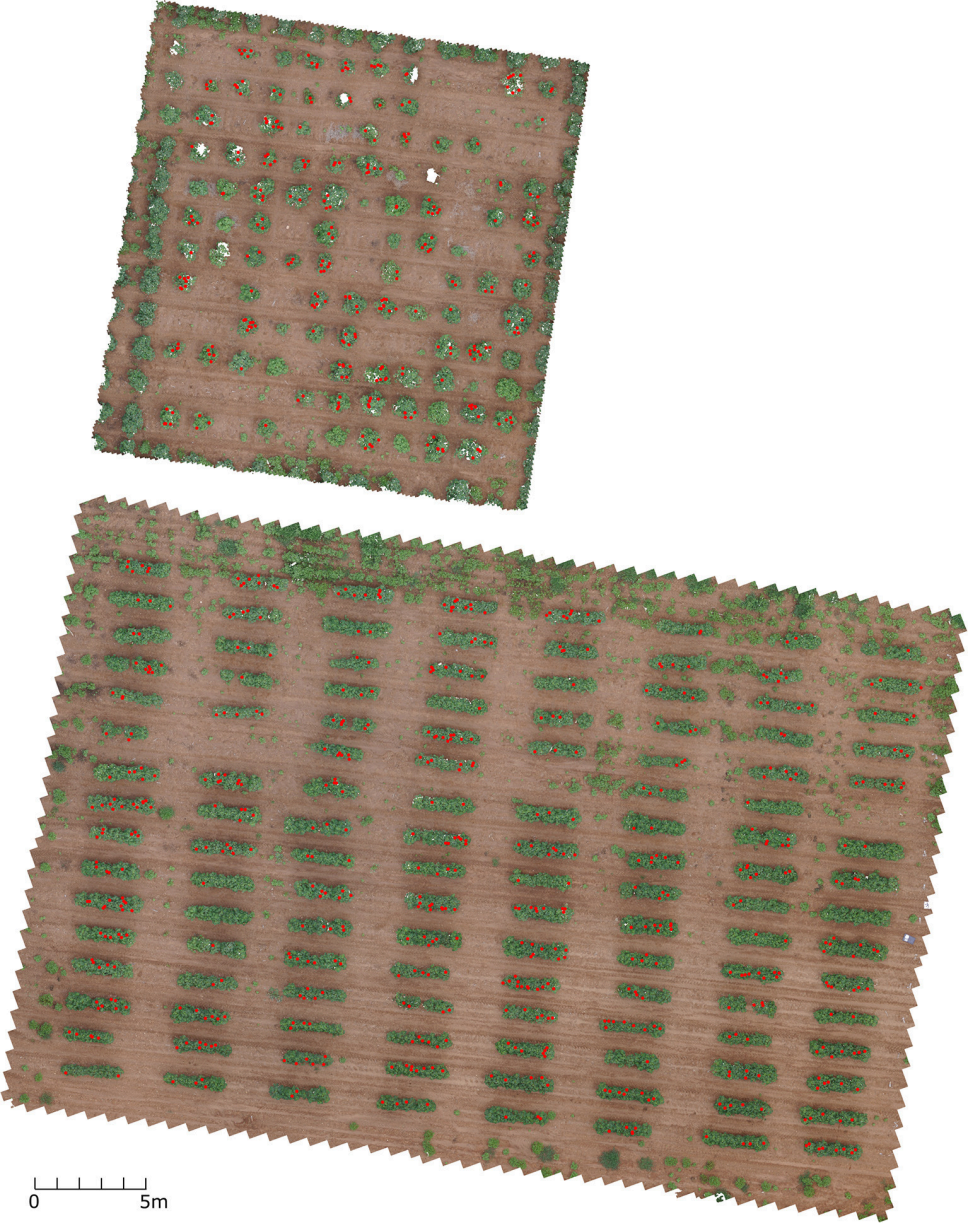
Top view of the two test fields with red dots indicating the flower locations using 8/12/2016 dataset. The image was rendered from dense point cloud.

Although the proposed bloom counting methodology usually provides an underestimated bloom count compared to manual counting, it has the advantage in throughput over manual counting. It saves manual labor and makes continuously monitoring the flowering possible. Without such throughput, it is impossible to continuously monitor the flowering stage and determine the flowering time, peak flowering time, and seasonal bloom count. For farmers and breeders, it is helpful to estimate the fiber yield because some studies have shown the fiber yield is correlated with seasonal bloom count (Heitholt, 1993, 1995). However, additional studies on how well the proposed methodology can estimate fiber yield are needed. The methodology is also helpful for differentiating the growth behavior among different genotypes, which can be used to select certain genotypes, such as short-season or long-season genotypes. Compared to other flower detection methods that are based on the percentage of flower pixels, the method proposed in this study can directly provide flower count without exploring the correlation between pixel percentage and flower count (Adamsen et al., 2000). The proposed method also can provide the locations of flowers as byproducts, which could be used to correlate with the cotton bolls (Figure 14).

To implement the proposed method, farmers and breeders can use commercial aerial photogrammetry systems or build custom systems to collect aerial images. The data processing pipeline can be used as long as the image quality (such as the ground resolution) meets the requirements. This study used small fields of 920 m^2^ (0.22 acre) and the data collection throughput is not enough for commercial farms or breeding programs with large fields. Although the throughput can be increased by increasing the flight altitude, the reduced ground resolution may not meet the requirement for the pipeline to correctly recognize cotton flowers. Alternative solution is to use high-resolution cameras to maintain the ground resolution when imaging at higher altitudes.

## Conclusion

This study developed a high throughput methodology for cotton bloom detection using aerial images, which can be potentially used to monitor cotton flowering over the season for cotton production management and yield estimation. The method generally underestimated the bloom count due to the inability to count hidden flowers, but the bloom count for the single plant layout was less likely to be underestimated than the multiple plant layout. The accuracy and completeness of the dense point cloud has an impact on the bloom count result, so generating a good dense point cloud can improve the results significantly. The bloom registration algorithm developed in this study was efficient in terms of runtime and was more prone to under-clustering but less prone to over-clustering. The trained CNN correctly classified more than 97% of the training and test images, and more than 90% of the potential flowers extracted from individual datasets. Since the false classification from the CNN can result in false bloom count, designing a robust CNN that can handle images taken under different field illumination conditions and cotton growth stages will be included in future studies. In addition, oblique imagery will be explored to improve the quality of the dense point cloud.

## Author contributions

RX: performed the data analysis and wrote the main manuscript text; CL and AP: designed the experiments and wrote the manuscript; YJ: designed plot layout and contributed to the data collection; SS and JR: contributed to the data collection and field management; All of the authors have read and approved the final manuscript.

### Conflict of interest statement

The authors declare that the research was conducted in the absence of any commercial or financial relationships that could be construed as a potential conflict of interest.
