# Supplementary material for: Aerial Images and Convolutional Neural Network for Cotton Bloom Detection
Source: Front Plant Sci. 2018 Feb 16;8:2235. doi: 10.3389/fpls.2017.02235 (PMC5820543; doi:10.3389/fpls.2017.02235)
Supplement: Supplementary file 1 [file Presentation1.PDF]

Supplementary figures

Figure S1 was generated from preliminary data using the plots from field 1 (single plot layout) that had both manual and image count. There are 70, 41 and 3 plots on 79, 86 and 107 days after planting, respectively. The overall trend from the image count matches with that from the manual count.

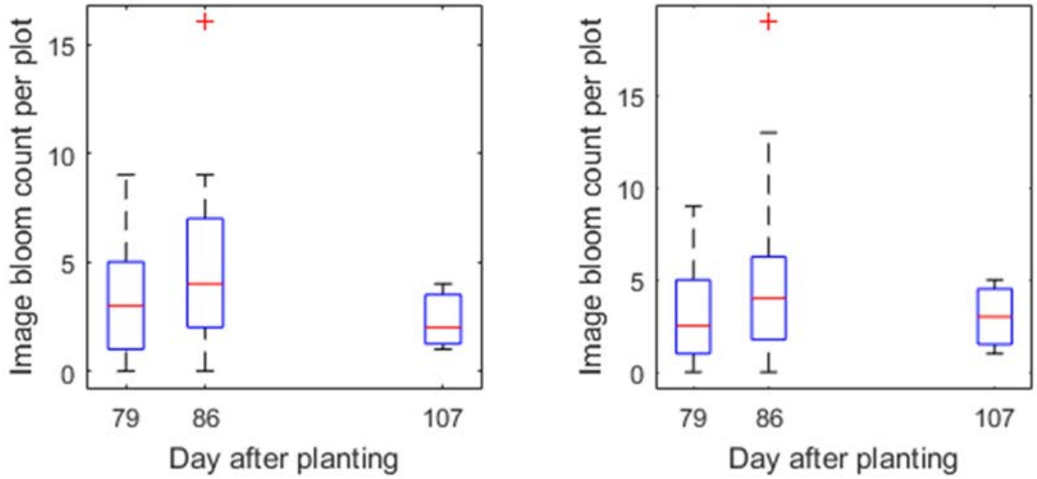

Figure S1 Boxplot of the flower count over time for field 1.

Field 2 has four genotypes and each genotype has 64 plots. The four genotypes are GA2011158 (genotype 1), GA2009037 (genotype 2), GA2010074 (genotype 3), Americot conventional (genotype 4).

Figure S2 to Figure S5 were generated from the preliminary data using plots from four genotypes in field 2 (10-feet plot) that had both manual and image count of blooms. There are 125, 9, 4 and 6 plots on 60, 67, 74 and 88 days after planting, respectively. The overall trend from the image count matches with that from the manual count. With limited plots, however, the underestimation could affect the trend. For example, image count on 74 days after planting has large underestimation, which makes the trend different from manual count on genotype 3.

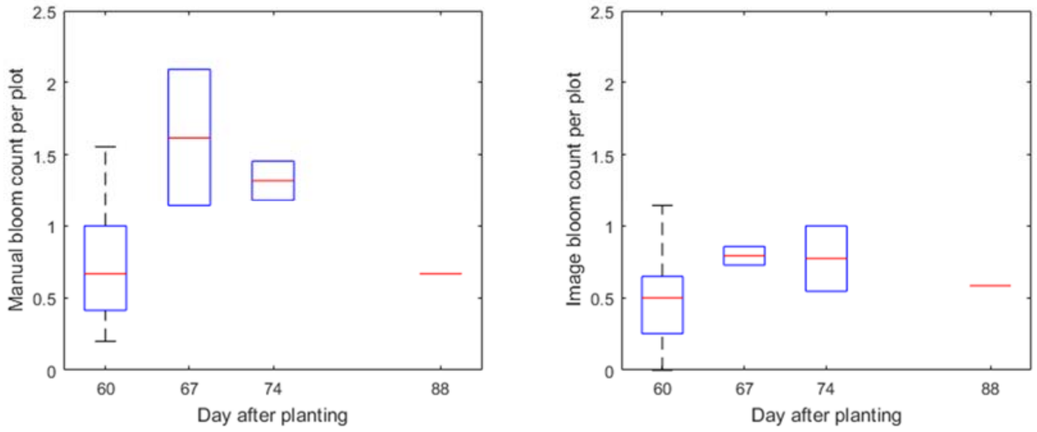

Figure S2 Boxplot of the flower count over time for field 2 for genotype 1. Sample size for DAP 60, 67, 74, 88 are 29, 2, 2, and 1, respectively.

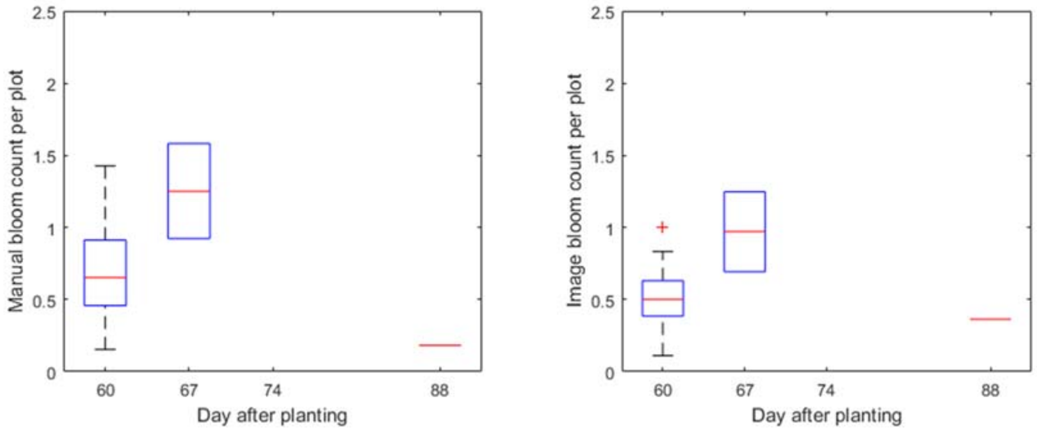

Figure S3 Boxplot of the flower count over time for field 2 for genotype 2. Sample size for DAP 60, 67, 88 are 32, 2, and 1, respectively.

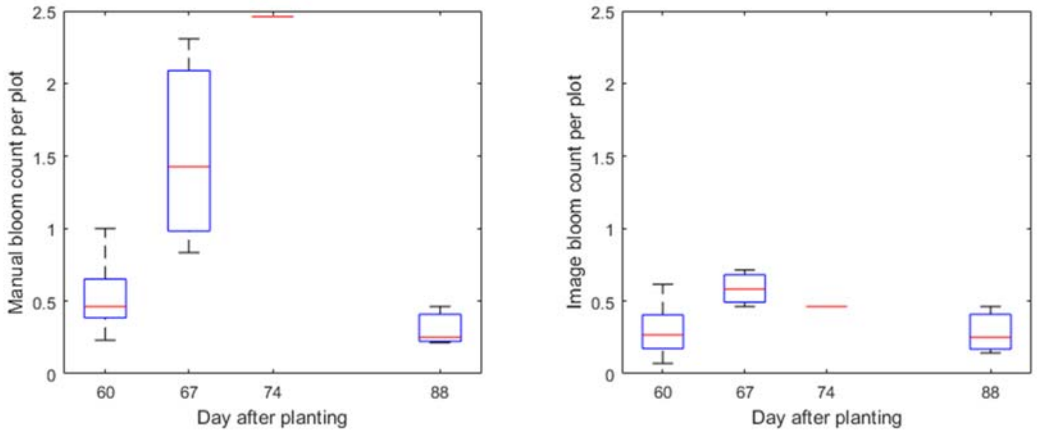

Figure S4 Boxplot of the flower count over time for field 2 for genotype 3. Sample size for DAP 60, 67, 74, 88 are 32, 3, 1, and 3, respectively.

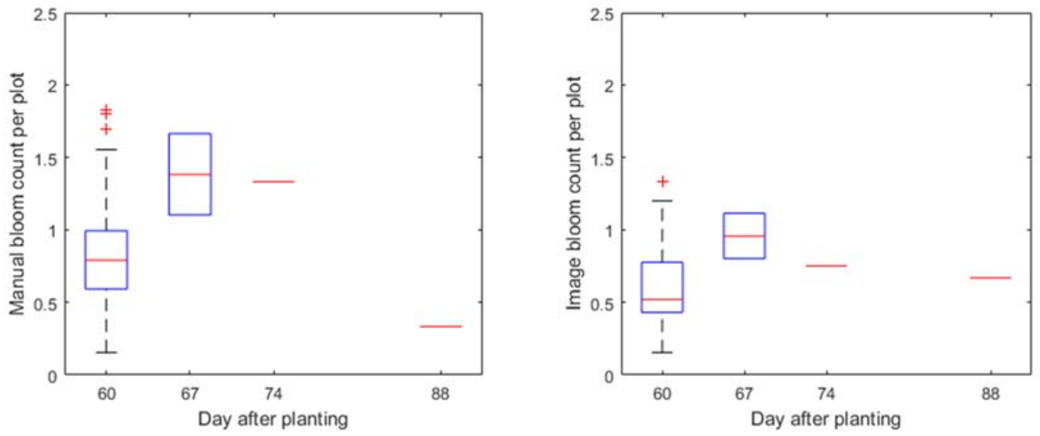

Figure S5 Boxplot of the flower count over time for field 2 for genotype 4. Sample size for DAP 60, 67, 74, 88 are 32, 2, 1, and 1, respectively.
